# What Is the Impact of a Context-Specific Training Program for Home-Based Carers? An Evaluation Study

**DOI:** 10.3390/ijerph17249263

**Published:** 2020-12-11

**Authors:** Mamare Adelaide Bopape, Tebogo Maria Mothiba, Hilde Bastiaens, Johan Wens

**Affiliations:** 1 Department of Nursing Science, School of Health Care Sciences, University of Limpopo, Polokwane 0700, South Africa; 2 Faculty of Health Sciences, University of Limpopo, Polokwane 0700, South Africa; tebogo.mothiba@ul.ac.za; 3 Department of Primary and Interdisciplinary Care, Faculty of Medicine and Health Sciences, University of Antwerp, 2610 Antwerp, Belgium; hilde.bastiaens@uantwerpen.be (H.B.); johan.wens@uantwerpen.be (J.W.)

**Keywords:** type 2 diabetes mellitus, home-based carers, context-specific, training program, evaluation

## Abstract

Introduction: In South Africa (SA), home-based carers (HBCs) play a crucial role at the community level for non-communicable diseases (NCDs) including diabetes mellitus (DM) public health care. The work of HBCs requires them to be knowledgeable about diabetes, and lack of knowledge has implications on their roles for the provision of health information and dietary advice. HBCs need to be provided with specific training to develop their knowledge and skills necessary to enable them to provide care to people with diabetes (PWD) because organizations need to benefit from a pool of well-trained HBCs. Therefore, a training program was developed to improve care for chronic conditions based on local needs. Aim: To implement and evaluate the training program for the HBCs for PWD in Ga-Dikgale village. Methods: HBCs working at Ga- Dikgale villages in four clinics—namely, Dikgale, Seobi Dikgale, Sebayeng, and Makotopong—participated voluntarily. Fifty-five (55) HBCs who attended the training program completed satisfaction survey tools, and furthermore, 45 HBCs completed both pre-training and post-training knowledge questionnaires. Training divided into two sessions which each lasted for two days was conducted. Satisfaction with the training, improvement of knowledge, and perceived impact on daily practice were evaluated using both qualitative and quantitative approaches. Results: Quantitative results indicate that 72% had poor knowledge of pre-training and only 9% post-training. They scored more in a post-test with the following differences: Post-test (mean = 6.00, SD = 1.26); pre-test (mean = 3.31, SD = 1.77). The *t*-test results indicated the difference to be significant, t = −9.241, *p* = 0.000. From the qualitative results, the themes that emerged during data analysis from group discussions were HBCs’ achievements from the training, challenges related to diabetes and diet, and suggestions for further training. Conclusions: A context-specific training increased diabetes knowledge among the HBCs for PWD. The results highlighted the importance of training in improving the knowledge of HBCs about the care of PWD. The improvement in diabetes knowledge among HBCs needs to be maintained and sustained to achieve major health benefits for PWD.

## 1. Introduction

Type 2 diabetes mellitus (T2DM) is a major form of diabetes mellitus (DM) and is continuously increasing in prevalence due to an increase in sedentary lifestyles and obesity. It was estimated that in 2018 there will be more than 500 million prevalent cases of T2DM globally and the prevalence will increase more in lower-income countries [1]. The number of people suffering from T2DM globally is also estimated to double from the current value in 25 years to come [2]. However, there are effective lifestyle modifications including weight loss, healthy dietary pattern, and physical activity that are the cornerstone in the prevention of T2DM [3]. Therefore, promotion of a healthy lifestyle should be emphasized to increase adherence and compliance to the lifestyle modifications more, especially for individuals at high risk. For those who are diagnosed with T2DM, an adequate treatment (including self-management), is important to prevent complications. There are many factors which influence the management and sustainability of diabetes self-care practices including social, political, environmental, cultural, and behavioral ones [4,5]. Therefore, multiple approaches that include education, social support, and community programs should be employed to address all these influences.

Since South Africa is faced with a rising burden of non-communicable diseases (NCDs) including T2DM, the country must expand its healthcare focus to include NCDs. The number of adults with diabetes has increased to 4.5 million people in SA [6]. Home-based carers (HBCs) are individuals who live within the community they serve, understand the culture, and speak the language of the people who live around them [7]. In South Africa (SA), home-based carers (HBCs) play a crucial role in the community level regarding NCDs including DM public health care [8]. They are uniquely positioned to collaborate with other health care providers to improve the quality of diabetes care and prevention in the communities [9]. 

Previously, HBCs were employed by non-governmental organizations (NGOs) to focus on NCDs, but currently, they are appointed by the Department of Health to function under the direct supervision of professional nurses to improve health outcomes [7]. HBCs can help prevent diabetes and control the disease and its complications through health education, lifestyle change, self-management, and social support. Implementation and evaluation of a training program for HBCs is a good practice after its development, to monitor its success. HBCs are effective in health-related practices, even though they lack knowledge in the care of people with diabetes (PWD) and need support from the nurses [10].

The work of HBCs requires them to be knowledgeable about diabetes, and the lack of knowledge has implications on their role as providers of health information and nutrition advice [11]. This role for HBCs as health information providers suggests that they are expected to act as sources of information and they should possess current and relevant information related to DM [8]. However, the lack of knowledge related to DM has resulted in HBCs providing information that is not always accurate, and this has highlighted the need for context-tailored training to standardize information given to PWD [8]. HBCs are important members of the diabetes care team and effectively assist in the management and care of diabetes in the community [12].

HBCs are increasingly included in the diabetic health care teams at the community level to provide care to people with diabetes (PWD). Since HBCs are increasingly being used for the management and care of chronic diseases including diabetes mellitus, it is important that training is available to increase their disease-specific knowledge [13]. Culturally tailored teaching will give the HBCs the relevant knowledge and tools to participate in the delivery of diabetes education to PWD [13]. They need to be provided with specific training to develop their knowledge and skills necessary to enable them to provide care to PWD because organizations need to benefit from a pool of well-trained HBCs [14]. 

The potential value of a training program must be tested for its success, and this cannot be possible without implementation and evaluation stages [15]. In a rural South African region (Ga-Dikgale, Limpopo Province) a context-specific training program was developed for HBCs. This paper describes the implementation and evaluation of this training program. 

### Objectives

The objective of this study was to implement and assess the HBCs’ training program, aiming for better prevention and self-care in PWD in Ga-Dikgale village. 

## 2. Materials and Methods 

### 2.1. Research Design

A convergent parallel mixed-method design was used to evaluate the impact of the implemented training program. Two sets of data were collected concurrently, but in parallel, using questionnaires for the quantitative part and focus group discussion for qualitative analyses. Simultaneous analysis of both quantitative and qualitative data was performed. The overall development, implementation, and evaluation of the training program was guided by the analysis, design, development, implementation, and evaluation (ADDIE) model. ADDIE model is an instructional design model consisting of the following five steps: Analysis, design, development, implementation, and evaluation (ADDIE) [16]. Each step of ADDIE feeds into the next step in the sequence.

### 2.2. Participants and Recruitment/Sampling

The participants were HBCs working at Ga-Dikgale villages under the four clinics namely: Dikgale, Seobi Dikgale, Sebayeng, and Makotopong. The HBCs were recruited from HBC centers, where they reported daily before going to see their patients; thus, they were approached and invited to participate in the study. HBCs’ participation in the study was voluntary, and they were allowed to terminate participation at any time without punishment. HBCs who attended the training (*n* = 55) were included during data collection to evaluate training satisfaction at the end of the session. Furthermore, the study included HBCs (*n* = 45) who participated in the pre-training assessment and during post-training data collection two months post-training. HBCs who were not part of pre-training assessment were excluded from participating. All participants provided written informed consent.

### 2.3. Training Implementation

A context-specific training program for HBCs who care for PWD was implemented at this phase. The design of the implemented training programme was based on the South African Qualification Authority (SAQA) guidelines [17]. Specific learning outcomes relevant to meet their job-related needs were developed based on the gaps between what the HBCs know, what they need to know, and their learning needs, as indicated during the situational analysis. The following modules were included in the training based on the HBCs’ training needs identified during training needs analysis: (1) Basic knowledge and classification related to diabetes mellitus which covers the concepts used for diabetes mellitus and care, normal regulation of blood glucose, types of diabetes mellitus, clinical differences between diabetes type 1 and type 2, signs and symptoms of diabetes mellitus, and risk factors of diabetes mellitus; (2) prevention and control of diabetes, which include early detection of diabetes by the FINDRISK tool, healthy diets, healthy food guide pyramid, plate model, role of physical activity in the prevention of diabetes, physical activities for diabetic patients, benefits of losing weight, avoidance of alcohol, and avoidance of tobacco use; and (3) management and prevention of complications by HBCs that covers the measures by HBCs to manage diabetes mellitus, the role of HBCs in assisting adherence to diabetic treatment, knowledge of glucose testing, assisting diabetic patients in treatment, complications of diabetes mellitus, hypoglycaemia, signs and symptoms, causes and prevention, diabetic foot problems, prevention of foot ulcer, amputation and wound dressing, and management and care of complications by HBCs [18]. The subject matter included was based on the previously developed material about T2DM and the research articles. Efforts were made to use the most recent information about DM. All the HBCs were provided with a hard copy of the training material to use before the training commenced.

#### Training Delivery

Dates for training were identified and arrangements were made with the managers of the HBC centers to release the HBCs for teaching. The training program was at Level 4 of the National Qualification Framework (NQF) with 11/2 credits. The contact sessions were divided into two for two groups of HBCs for interactive facilitation with different facilitators. The training lasted for two days with eight-hour sessions per day. Training took place at a central venue away from the HBCs’ workplace to avoid disturbances during training and to enhance learning. The training program was delivered by the professional nurse (researcher, a lecturer with a Master’s degree), psychologist (lecturer with a Master’s degree), dietician (a lecturer with a Master’s degree), and pharmacist (lecturer with a Master’s degree).

### 2.4. Data Collection

Evaluation of both quantitative and qualitative data was guided by the Kirkpatrick four-level evaluation model [19].

#### 2.4.1. Reaction (Level I)

Reaction refers to the way in which HBCs measure their satisfaction with the training [20,21]. Fifty-five (55) HBCs who attended the training were given evaluation tools to complete at the end of each session to assess their reaction on the training. Evaluation indicators included training objectives, topic, content, venue, time, environment, facilitator’s knowledge and preparedness, training manuals, the benefits of training on the job, and what they think should have been included in the training. HBCs were asked to rate these indicators on a 3-point Likert scale. Additionally, HBCs were also asked to write suggestions on the training in the comment box. HBCs were given 45 min to complete the evaluation tools at the end of the session.

#### 2.4.2. Learning (Level II)

Learning in this study referred to the extent to which the attitudes of the HBCs changed, their knowledge increased, and their skills broadened post-implementation of training [22]. The evaluation of learning measured what the participants had learned and gained from the training program [22]. The questionnaire consisted of three sections: Section A: Demographic data, section B: Self-evaluation, and section C: Tested knowledge. Section A consisted of 7 (seven) multiple-choice questions used to assess some biographic characteristics of the HBCs. Section B consisted of 13 statements of self-evaluation tools with a 5-point Likert type scale ranging from 1 (strongly disagree) to 5 (strongly agree) aiming to assess actual common practices of HBCs towards people with diabetes. Section C consisted of 10 multiple choice questions to assess HBCs’ actual knowledge related to diabetes. A scoring system was created for these knowledge questions by giving one score for each correct answer and a 0 score for incorrect, uncertain, or missing answers. Knowledge questions were adopted from diabetes knowledge questionnaires [23,24] and selected based on the results obtained from during the situational analysis phase.

Data from the HBCs who did not take part in the baseline assessment and post-training evaluation were not included in the summary. For this phase, section C of the questionnaire was the major focus to evaluate knowledge of HBCs post-training. HBCs were asked to complete questionnaires and only 45 questionnaires were collected for data analysis to compare with pre-training analysis.

In addition to the quantitative evaluation, 2 follow-up focus group discussions were conducted two months after completion of the training program for the qualitative part of the study. The focus group discussions were conducted to evaluate the last two levels of Kirkpatrick, behavior, and results. The focus of the behavior level was on the exploration of the extent to which the knowledge and skills acquired in the training were applied back at work [22]. The results level focused on the realization of aimed objectives and change in productivity after training [22].

The researchers (MA and TM) guided the focus group discussions. Each focus group consisted of 14 and 15 HBCs, respectively. The interview process was explained beforehand and only started when participants had given voluntary consent to their participation. An opening question, “Have you applied the acquired knowledge in your daily work? If so, give examples”, was asked to the HBCs to direct the focus group discussion followed by probing questions. Probing questions were guided by the responses to the initial question. The HBCs reported on their changes in behavior when they returned to work after attending the training. HBCs were encouraged to report on the challenges they experienced in the application of knowledge and skills learned during training. They were also encouraged to give suggestions for training improvement. The focus group discussions were voice recorded and lasted for 1 h 30 min. The recorded data were then transcribed verbatim in preparation for data analysis.

### 2.5. Data Analysis

The Statistical Package for the Social Sciences (SPSS) 25 (IBM Corp., Armonk, NY, USA) was used to analyze descriptive statistics, mean differences before and after training a repeated-measure *t*-test for the level of significance, and MS Excel Office was used for the item analysis of the knowledge test questions. For the knowledge test questions, responses were coded a “0” incorrect answer and “1” correct answer. Descriptive statistics were presented as individual scores, means, standard deviations, and percentages. The participants’ mean scores before and after the training were compared using a paired sample *t*-test. The level of significance was set at 0.05. Analysis of all the knowledge questions was also done to calculate item difficulty, with a discrimination index to determine how well a question distinguished between students who performed well and who did not perform well. Average total score per item = pg(x) of those HBCs that answered the question correctly were also calculated. Item difficulty (px) also called *p*-value was calculated to determine the difficulty of each question using the following formula: px = total number of correct answers divided by the number of people who answered the question (above 0.8 = easy, 0.3–0.8 = medium, and under 0.3 = hard). The discrimination index ranged from −1 to 1. The ranges were defined as follows: 0.4 and up = good, 0.3–0.39 = satisfactory, 0.2–0.29 = middling, and 0.19 and below = poor. The correlation was also calculated to indicate the correlation between an individual score on the question and their total exam score with the following ranges: 0.35 and up = good, 0.25–0.35 = satisfactory, 0.15–0.25 = middling, 0.15 and below = poor.

Qualitative data obtained through focus groups were analyzed using a thematic analysis method [25]. The audio recordings were transcribed verbatim into a text by the researcher in preparation for data analysis. The researchers (M.A. and T.M.) read through all the transcripts and familiarized themselves with the entire body of data. After that, the researchers generated initial codes from each segment of data found to be relevant to the research question. The researchers examined the codes in search for themes, and some of them fit into the themes. Data associated with each theme were reviewed and considered as to whether they supported the themes. Finally, the themes were defined and named, and the report produced.

## 3. Results

In total, 55 HBCs attended a two-day training program delivered by two facilitators. Facilitators involved in the training included a researcher who was also a professional nurse knowledgeable about DM, a dietician with expertise related to DM, a pharmacist knowledgeable with expertise about diabetic treatment, and a psychologist.

### 3.1. Satisfaction with the Training (Reaction Level)

The majority 49/55 (89%) of the HBCs who attended training agreed that the training objectives were clearly defined. All of the HBCs 55/55 (100%) agreed to the following: The topics covered were relevant, the materials distributed were helpful, and the facilitators were knowledgeable about the training topics. Only 40/55 (73%) HBCs agreed that the time allocated tor training was sufficient (Table 1).

### 3.2. Biographic Characteristics of HBCs

Approximately 27/45 (60%) of the HBCs’ had between 5 and 10 years of experience (mean 9 years), 5/45 (11%) less than 5 years and 13/45 (29%) had more than 10 years’ experience working as HBCs. Forty (40/45 (89%)) of the respondents had secondary education and 5/45 (11%) had tertiary education. Forty (40/45 (89%)) had never attended an in-service training on diabetes and only 5/45 (11%) attended. Just 4/45 (9%) HBCs indicated they felt competent about caring for people with diabetes, whereas 17/45 (38%) indicated being somewhat competent, and 24/45 (53%) not competent at all.

### 3.3. Quantitative Evaluation of the Learning Level

Table 2 shows the item analysis results of the knowledge test questions. The item difficulty in the pre-test indicates that four out of 10 questions were considered hard, whereas the results of the post-training test indicate that the majority of the questions’ difficulty level was average (between 0.3 and 0.8) and only one was easy. The discrimination index of post-training results indicates that only 4 questions indicated good discrimination, whereas we had 6 in the pre-test results. Three questions in the post-training test indicated satisfactory discrimination index compared to only one in the pre-training test. All the questions in the post-training test indicated a poor correlation between individual question scores and all the correct scores, and only one question in the pre-training test indicated a good correlation.

Table 3 presents the performance of HBCs in the pre- and post-knowledge tests for each variable. The number of correct answers in the post-test was higher than those in the pre-training for each question. This suggests that the knowledge of HBCs in the post-training was better than in the pre-test after undergoing training on the care of diabetes for 2 days. The pre-mand post-training knowledge scores of HBCs showed that 72% of the HBCs had poor knowledge pre-training and 80% had average knowledge post-training (Table 3). Post-training scores were significantly higher than the pre-training scores, indicating an increase of knowledge. From the results shown in Table 3, the mean for post-training scores was higher than those of the pre-training. This demonstrates that the HBCs’ performance in post-training assessment was better than pre-training assessment after taking the diabetes training.

The results demonstrated that the respondents scored more in a post-test with the following differences before and after training: (Mean = 6.00, SD = 1.26) than in a pre-test (mean = 3.31, SD = 1.77). A repeated-measure *t*-test indicated the difference to be significant, *t* = −9.241, *p* = 0.000 (Table 4). There was strong evidence that (*t* = −9.241, *p* = 0.000) the training of HBCs improved their knowledge.

### 3.4. Qualitative Evaluation of the Behavior and Results’ Levels

Two focus group discussions that explored the impact of the training on HBCs’ daily practice were conducted. Table 5 provides a summary of the results on behavior and result levels of HBCs for PWD post-training.

The narrative that follows deals with the themes and sub-themes that emerged from the focus group discussions with HBCs. The findings represent the extent to which HBCs apply the knowledge and skills acquired in the training and the change in productivity after training regarding each of the sub-themes of the main themes. The findings are presented below and are supported with the participants’ quotations.

### 3.5. Theme 1: Benefits Acquired from Training

The findings described the participants’ benefits about the information gained from the training, which includes the following: HBCs’ reported enhanced knowledge/application of the learned knowledge during provision of care and HBCs’ perceived change in productivity.

#### 3.5.1. Sub-Theme 1.1: Enhanced Knowledge and Skills towards Their Work Obtained

The study findings indicate improved HBC knowledge after training because they gave advice to patients and anyone asking about diabetes health education with confidence. They also gave patients at the clinics’ health education about diabetes.

[HBC]… “Before I attended the training, I had a problem when the patients ask me about diabetes like diabetic diet, but now with the information I received from the training, I give my patients and anyone who asks about diabetes health education with confidence based on the information received from the training”.

[HBC3]… “Before we attended the training I used to go to the clinic and sit with the patients and listen to the nurses when they give health education. But since I attended the training, I go to the clinic and give patients health education about diabetes”.

#### 3.5.2. Sub-Theme 1.2: HBCs Perceived Change in Productivity

Based on the data collected from the HBCs, it was indicated that there was a change in their attitude towards work and productivity. The participants managed to convince PWD to take treatment at the correct time (adherence), gave information on diabetes, diet, and physical activity, and this resulted in controlled glucose level, wound healing, and improvement of the condition. This was supported by the following participants’ quotes:

[HBC]… “I think I am more patient with my patients now as compared to before I attend[ed] training. The doctor changed her oral treatment to injection and the patient decided to stop taking treatment and went to the traditional healers. I have decided that I will talk to her and try to convince her to take medication. I have talked to the patient and convinced her to start using her injections. After some time, the patient thanked me for the support that I give her”.

[HBC]… “Adherence… I advised the patient who was not taking treatment well that she was supposed to take treatment at the right time. Because of the information I have, I managed to convenience the patient to take the treatment at the right time and told her about the benefits of taking the treatment at the right time. The patient listened to me and after some time she informed me that she was from the clinic and they [have] tested her sugar level, and it is controlled”.

[HBC]… “We had a patient with a wound and we were dressing for a long time. After the training, we sat him down and advised him about diabetes and diet, exercises, and taking treatment on time. We emphasized mostly lifestyle and, fortunately, the patient listened to us. As I speak now, we are no longer dressing the wound; it has healed, and the blood glucose is controlled. We have realized that you can take medication, but lifestyle is the key to the management of diabetes. The person can now wear the shoe and he was unable to wear it before. We also advised him to not to walk outside without shoes”.

[HBC]… “A relative [who was] also a diabetic patient/client was unable to use his legs properly and I have asked to see the treatment. When I check[ed] the treatment, I found that there was no instruction on the treatment packages. The patient was taking all the treatment once per day. I advised the patient to ask them to write instruction[s] on the packaging. The person is taking the treatment well as prescribed and he is now better, and the hands are becoming better”.

### 3.6. Theme 2: Challenges Experienced by HBCs Post-Training during Care Provision

Although HBCs attended training and are knowledgeable about diabetes care and management at the community level, they still encounter some problems during the provision of care. It was discovered from the data collected that they still face challenges related to a diabetic diet, exercises, and treatment which are related to patients’ attitudes and socio-economic status.

#### 3.6.1. Sub-Theme 2.1: PWD’s Attitude Related to Diet

HBCs are experiencing challenges related to PWD complaining about food affordability and that they do not eat vegetables and beans.

[HBC]… “Yes, we were taught [about] and understood diabetes, and when we arrive[d] at home we had to tell the patients about diabetic diet, but the challenge was that they were complaining about the affordability of food because they are responsible for the whole family”.

[HBC]… “One person I advised about diet told me that he eats meat and he cannot eat vegetables because he is not a rabbit. He cannot eat beans because they do not eat seeds”.

#### 3.6.2. Sub-Theme 2.2: PWD’s Attitudes Related to Diabetic Treatment

The findings indicated that the HBCs experience challenges in cases where their patients collect treatment from the clinic and throw it away, and some take treatment at different times.

[HBC]… “The patient takes treatment at the clinic and then throw[s] them away. The problem is that blood glucose will not be controlled”.

[HBC]… “I have [a] patient taking treatment at different times. So, I have advised him to take the treatment at the same time and then told him about the importance of taking treatment at the same time”.

#### 3.6.3. Sub-Theme 2.3: PWD’s Attitude Related to Physical Activity

The results have shown that HBCs patients encounter patients who sit at the same place the whole day without engaging in physical activity and refuse to walk with support.

[HBC]… “I have a patient who sits at the same place the whole day. They gave her food and after eating they come and take the dishes. I have advised her about exercising and she told me that if [she fell] while walking and die[d] what you will say”.

[HBC]… “I have a patient who has difficulty in walking. When I go to the place, I offer support and encourage her to walk. I advised the family members to support her to walk. Then from there when I go there, I found her outside and she reported that she walked herself outside, until the child told me that they ha[d] carried her outside. So, when I tried to discourage her, she said if it was your mother, were you going to tell her the same thing that you are telling me”.

### 3.7. Theme 3: HBCs Post-Training Suggestions for Further Training

HBCs indicated that the training was very helpful; however, there were things that they suggested had to be added in the future training so that they would be able to provide quality care to PWD. The HBCs indicated the need to include administration of injections, demonstrations on glucose testing, and measuring of injections.

#### 3.7.1. Sub-Theme 3.1: Suggestions Related to Training on Injectable Treatment

The HBCs demonstrated the need to be trained on injectable treatment because patients who use injections ask them to measure, withdraw, and inject their medications.

[HBC]… “I have a patient who uses injections and when I arrive, she asks me to assist to inject her. My problem is I cannot because I was not taught how to inject. It would have been better if training [could] include teaching us to inject so that we can help our patients fully”.

[HBC]… “If we are not taught on how to inject, I suggest that we are shown how to measure the injections so that we can withdraw for our patients or check if they have withdrawn the correct amount”.

#### 3.7.2. Sub-Theme 3.2: Suggestions Related to Training on Glucose Testing

The data collected during focus group discussions demonstrated that HBCs suggested including demonstrations of glucose testing with machines, and practicing glucose testing with each other during training.

[HBC]… “I think it would have been better if we were having machines to test glucose and all of us ha[d] an opportunity to practice testing glucose with the machine because our patients expect us to assist them with glucose testing.

[HBC]… “It will be better when we test each other’s glucose using a machine and that will maybe give us a chance to test our glucose. That way we will be learning, and we can therefore go and assist our patients with what we have learned”.

## 4. Discussion

This paper focused on the teaching and evaluation of a training program for HBCs who care for PWD by following the last two stages of the ADDIE model (implementation and evaluation). The evaluation was based on Kirkpatrick’s four levels of evaluation model using both quantitative and qualitative approaches. The evaluation results indicated that HBCs were satisfied with the training program and their test results showed a significant improvement in their knowledge and skills after completion of the training. According to the evaluation focus group discussions, they applied the knowledge and skills they learned in their daily work.

The initial evaluation of the training indicated that HBCs were satisfied with the training. In support of our results, another study indicated favorable reactions to the training program which coincided with the increase in knowledge and confidence of HBCs during provision of care to PWD [26]. The HBCs received the training program positively, and some indicated that the time was not sufficient for training, meaning that it could be prolonged for them to learn more. The HBCs were satisfied with the content of the training, the venue, training objectives, training topics and activities, facilitators, training methods, and training manuals [22,27]. The results of satisfaction indicated that the training was well received by the HBCs.

Concerning learning, in concurrence with other studies, the study findings indicated a significant increase in the diabetes knowledge of the HBCs post-training [10,26,27,28,29]. Pre-training results indicated that HBCs lacked knowledge about diabetes mellitus, types of diabetes mellitus, the ideal range for blood glucose, signs and symptoms of diabetes mellitus, diabetes diet, diabetes treatment, prevention and control of diabetes mellitus, the complications of diabetes mellitus, and management of diabetic complications. This was very much concerning as the HBCs were expected to assist their clients with the prevention and management of diabetes at the community level. Therefore, there is a need to increase knowledge and skills of HBCs if they are to get a real place in the prevention and care of NCDs as a whole in the South African healthcare system.

The study findings indicated an increase in post-training evaluation scores as compared to the pre-training evaluation scores [30]. Post-training scores in support of other studies were higher than pre-training scores, indicating that they learned more about diabetes during training [10,31]. Using item statistics was a limitation for this study as it was recommended in studies with larger samples. However, the test assisted in discovering that the pre-test might have been too complicated for the HBCs. This is because of their limited knowledge, as foreseen, which might have major implications for the quality of care they delivered. Therefore, item statistics analysis assisted in the identification of item difficulty and discrimination index, which assisted in concluding HBCs’ knowledge post-training.

Regarding behavior and results levels that measure a change of behavior and performance, it was indicated that the evaluation needed to be done after a long time. However, in this study, evaluation was done two months after training and the findings indicated a vast change in behavior and performance. The study findings indicated a change in the behavior of HBCs in their job where they were capable of giving health education about diabetes to their patients and those attending clinics on a daily basis. Concurrent with other studies, the study indicated the role of the training in improving the knowledge of HBCs about diabetes care and management [26]. In support of the study findings, the study conducted by Gao et al. [27] indicated that the participants applied the acquired knowledge in various aspects of their daily work post-training. The HBCs needed to gain self-confidence after their training, which indicates that they will love their work more.

The limitation of this study was that the findings were drawn from a small population and from a limited rural area of the Limpopo Province in South Africa. Therefore, the study participants may not represent the general population. However, the program implementation provided a significant contribution to HBCs’ knowledge in the limited local context, which is useful for the care and management of PWD, and needs to be implemented and evaluated in more clinics. Possibly, different areas and clinics with HBCs could have different needs.

HBCs met the needs of PWD by providing diabetes information and support. Assessing acquired knowledge and skills after training is important to ensure that they possess relevant and necessary information during the provision of care to PWD. Therefore, it is necessary for HBCs to continuously attend refresher training in diabetes management so that the knowledge and skills learned are not lost. HBCs need to allocate nurses specifically to continuously evaluate their knowledge and skills and support them. If HBCs receive training in the management and control of diabetes, major public health benefits are likely to be achieved in many resource-constrained settings with similar networks of HBCs at the primary care level of the health system. Training, both in the beginning and throughout the lifespan of the HBCs time, will help to further their competency and allow them to feel supported in providing services while also ensuring the devotion of evidence-based service delivery.

## 5. Conclusions

In conclusion, it was discovered from this study that a context-specific training program implementation has an impact on HBCs’ knowledge, skills, and behavior that will ultimately benefit PWD and the health sector. To understand the impact of training implementation, the evaluation of HBCs’ reaction to the training, learning, and behavior after training was implemented. The training was well-received by HBCs as evidenced by positive feedback. The training implementation resulted in a contextual increase in diabetes knowledge, skills, and behavior change among the HBCs for PWD. The improvement in diabetes knowledge, skills, and behavior change among HBCs needed to be maintained and sustained to achieve major health benefits for PWD. If all HBCs received context-specific training about diabetes, especially before the commencement of their job, it would be easier for them to provide care to PWD with the relevant information and skills. Possibly, different areas and clinics with HBCs could have different needs; therefore, further studies could be carried out in the different clinics to understand their training needs for program implementation to enhance knowledge and skills of HBCs for PWD.

## Figures and Tables

**Table 1:** A training evaluation tool.

Items	Agree		Neutral		Disagree	
	*n*	%	*n*	%	*n*	%
1. The objectives of the training were clearly defined.	49	89	4	7	2	4
2. Participation and interaction were encouraged.	52	95	3	5	0	0
3. The topics covered were relevant to me.	55	100	0	0	0	0
4. The content was organized and easy to follow.	54	98	1	2	0	0
5. The materials distributed were helpful.	55	100	0	0	0	0
6. This training experience will be useful in my work.	53	96	2	4	0	0
7. The facilitators were knowledgeable about the training topics.	55	100	0	0	0	0
8. The trainer was well prepared.	54	98	1	2	0	0
9. The training objectives were met.	50	91	3	5	2	4
10. The time allocated for the training was sufficient.	40	73	2	4	13	23
11. The training venue and facilities were adequate and comfortable.	50	91	4	7	1	2

**Table 2:** Item analysis.

Items	Difficulty		Pg(x)		Discrimination Upper		Discrimination Lower		Discrimination Index		Correlation R(x)	
	Pre	Post	Pre	Post	Pre	Post	Pre	Post	Pre	Post	Pre	Post
C1	0.3	0.5	4.6	6.0	10	10	1	6	0.7	0.3	0.1	0.0
C2	0.2	0.6	4.4	5.9	7	10	2	6	0.4	0.3	0.4	0.0
C3	0.2	0.4	4.8	5.8	7	6	0	4	0.5	0.1	0.0	0.0
C4	0.7	0.8	3.6	5.8	10	11	7	10	0.2	0.0	0.0	0.0
C5	0.1	0.5	4.2	6.1	3	10	1	7	0.1	0.2	0.0	0.0
C6	0.3	0.5	4.1	6.2	6	10	2	4	0.3	0.5	0.0	0.0
C7	0.4	0.5	4.1	6.4	7	8	1	2	0.5	0.5	0.1	0.0
C8	0.0	0.5	5.0	6.1	2	8	0	3	0.1	0.4	0.0	0.0
C9	0.4	0.5	4.2	6.3	8	9	1	2	0.5	0.5	0.1	0.0
C10	0.3	0.6	−0.1	6.1	8	8	1	4	0.5	0.3	−0.1	0.0

**Table 3:** Pre- and post-training knowledge scores.

Questionnaire Items	Pre-Training Correct Answers		Post-Training Correct Answers		Difference
	*N*	%	*n*	%	
C1. What is an ideal range for blood glucose (sugar) levels for a person with diabetes (PWD)?	17	38	25	56	+8
C2. What is the correct diet for a patient with diabetes?	12	27	27	60	+15
C3. What are the characteristics of type 2 diabetes mellitus?	10	22	20	44	+10
C4. How often should people with diabetes exercise or be physically active?	32	71	39	87	+7
C5. Diabetic people need a medical check-up of their eyes/nerves and kidney function at least: (Frequency)	5	11	26	58	+21
C6. If a PWD has a hypo (low blood glucose level) reaction, s/he should …	15	33	23	51	+8
C7. When should a well-controlled PWD always check urine for ketone?	19	42	23	51	+4
C8. What effect does illness (for example, a “sick day”) have on a PWD’s insulin requirements?	4	9	23	51	+42
C9. Why is it necessary that PWD pay special attention to proper care of their feet?	19	42	25	56	+14
C10. A PWD has just received a minor abrasion on the left leg. What should be done to treat the abrasion?	16	35	28	62	+27
Knowledge level	Range	Frequency		Percentage	
		Pre	Post	Pre	Post
Poor knowledge	0–4	38	4	72%	9%
Average knowledge	5–7	15	36	28%	80%
Good knowledge	>7	0	5	0	11
Total		53	45	100%	100%

**Table 4:** Paired samples statistics: Means.

	Mean	Std Deviation	C.I		*p*-Value
Pre-test	3.31	1.769	2.78	3.84	0.00
Post-test	6.00	1.261	5.62	6.38	

**Table 5:** Themes and sub-themes reflecting behavior and results levels of the home-based carers (HBCs) post-training.

Themes	Sub-Themes
Benefits acquired from training	1.1 Enhanced knowledge and skills towards their work obtained 1.2 HBCs perceived change in productivity
2. Challenges experienced by HBCs post-training during care provision	2.1 PWD’s attitude related to diet 2.2 PWD’s attitude related to diabetic treatment 2.3 PWD’s attitude related to physical activity
3. HBCs’ post-training suggestions for further training	3.1 Suggestions related to training on injectable treatment 3.2 Suggestions related to training on glucose testing
